# Custom-Made Alumina Ceramic Total Talar Prosthesis for Idiopathic Aseptic Necrosis of the Talus: Report of Two Cases

**DOI:** 10.1155/2017/8290804

**Published:** 2017-05-29

**Authors:** Ichiro Tonogai, Daisuke Hamada, Yuhei Yamasaki, Keizo Wada, Tomoya Takasago, Takahiko Tsutsui, Tomohiro Goto, Koichi Sairyo

**Affiliations:** Department of Orthopedics, Institute of Biomedical Science, Tokushima University Graduate School, 3-18-15 Kuramoto, Tokushima 770-8503, Japan

## Abstract

Two women aged 65 years and 78 years presented to our center with idiopathic necrosis of the talus. In both cases, imaging examinations showed collapse and sclerotic changes of the talar body caused by the necrosis. Both women underwent resection and placement of a third-generation custom-made alumina ceramic total talar prosthesis. Immobilization of the ankle in a short-leg cast for 3 weeks was followed by early rehabilitation. One year and 6 months after surgery, both women were able to walk without pain. Their Japanese Society for Surgery of the Foot ankle-hindfoot scale scores improved from 22 and 29/100 points to 90 and 95/100 points, respectively. To the best of our knowledge, the successful treatments of these two rare cases of idiopathic necrosis of the talus are among only a few reported cases of using a third-generation alumina ceramic prosthesis.

## 1. Introduction

The blood supply to the talus is relatively poor. The supply through the periosteum is limited because approximately 60% of the surface of the talus is covered with articular cartilage [1] and the talus has no muscular or tendinous attachments [2]. The talus is, therefore, at increased risk of necrosis.

Treating necrosis of the talus is difficult, especially when the necrotic area is extensive. Conservative treatment requires a long non-weight-bearing period and is likely to have an unsatisfactory outcome [3]. Surgery such as tibiocalcaneal arthrodesis results in shortening of the leg and loss of function [4].

A total talar prosthesis that was developed and refined in Japan has had excellent postoperative outcomes [5–7]. However, to our knowledge, there are few reports of the use of a third-generation alumina ceramic talar prosthesis for necrosis of the talus. Here, we report two rare cases of idiopathic necrosis of the talus successfully treated by resection and placement of a custom-made alumina ceramic talar prosthesis.

## 2. Case Report 

Both patients gave their informed consent for publication of this report.

### 2.1. Case 1

**Figure 1:**
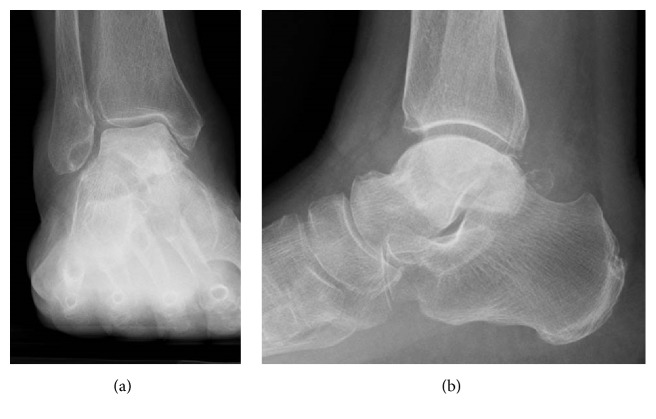
Preoperative radiographs showing sclerotic change of the talar body in anteroposterior (a) and lateral (b) views.

**Figure 2:**
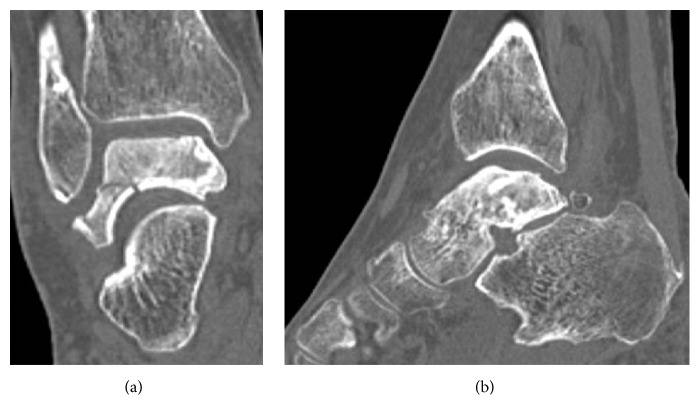
Preoperative CT scans showing sclerotic change and collapse of the talar body in coronal (a) and sagittal (b) views.

**Figure 3:**
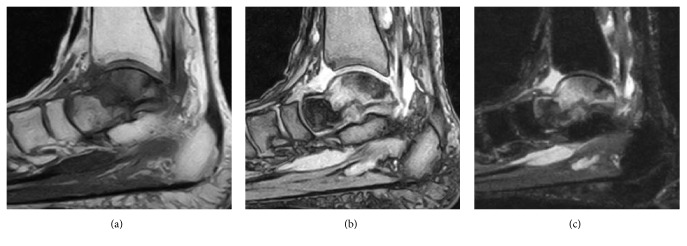
Preoperative MRI scans showing heterogeneous low signal intensity in the talar body on a T1-weighted image (a), low signal intensity in the talar neck and weight-bearing area of the talar body on a T2-weighed image (b), and heterogeneous high signal intensity in the talar body on a STIR image (c).

A 65-year-old woman with hypertension was referred to our department with a 7-month history of right ankle pain. She had no history of corticosteroid use, trauma, or alcohol abuse. She had difficulty walking (video 1, in Supplementary Material available online at https://doi.org/10.1155/2017/8290804) and used a T cane even when walking at home. Physical examination revealed swelling and tenderness over the anterolateral and anteromedial aspects of the right ankle. Dorsiflexion of the right ankle was 5° and plantar flexion was 20°. Her Japanese Society for Surgery of the Foot (JSSF) ankle-hindfoot scale score was 22/100 (pain 0/40, function 22/50, and alignment 10/10) and her visual analog scale (VAS) score for pain was 9/10. Radiography revealed peripheral sclerosis in the talar body (Figures 1(a) and 1(b)) and computed tomography (CT) revealed collapse and sclerotic changes in the talar body (Figures 2(a) and 2(b)). Magnetic resonance imaging (MRI) showed an expansive talus lesion. T1-weighted images showed heterogeneous low signal intensity in the talar body (Figure 3(a)). On T2-weighted images, signal intensity in the talar neck and weight-bearing area of the talar body was low (Figure 3(b)). Short time inversion recovery (STIR) images showed heterogeneous high signal intensity in the talar body (Figure 3(c)). The diagnosis was idiopathic necrosis of the talus, which was treated using a custom-made alumina ceramic total talar prosthesis fabricated by Kyocera Co., Ltd. (Kyoto, Japan) using mirror CT images of the contralateral talus. The prosthesis had appropriate articulation in the talocrural, subtalar, and talonavicular joints to allow stability of the prosthesis within joints.

A tourniquet inflated to 250 mmHg was placed around the thigh. The talus was exposed using an anterior approach between the tibialis anterior and extensor hallucis longus tendons. Soft tissue attached to the talus was dissected and the entire talus was removed piece by piece. The superficial layer of the deltoid and calcaneofibular ligaments remained to be untouched. The talar prosthesis was inserted using the instrumentation and techniques recommended by the manufacturer. The prosthesis was placed so that the superior aspect of the prosthesis articulates with the facet of the distal tibia, the inferior aspect of the prosthesis articulates with the superior facet of the calcaneus, and the anterior aspect of the prosthesis articulates with the proximal facet of the navicular. Ligament reconstruction was not performed because the prosthesis was in a stable position between tibia, calcaneus, and navicular once the prosthesis was inserted. The anterior ankle capsule, which was maintained as much as possible, was sutured to prevent anterior migration of the prosthesis after surgery. The wound was closed after correct positioning and alignment of the talar prosthesis was confirmed by radiographic examination. There were no intraoperative complications.

**Figure 4:**
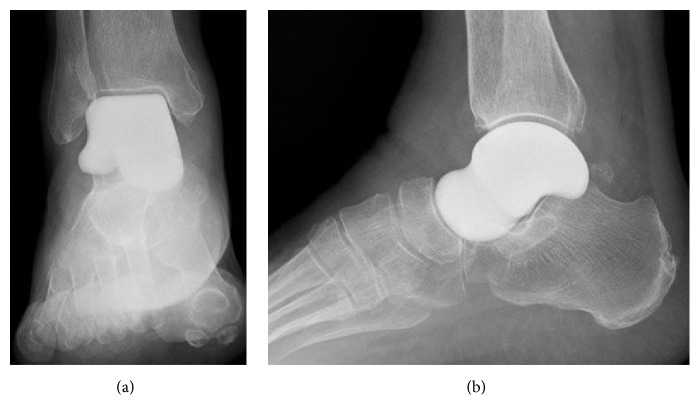
Postoperative radiographs showing the outcome of replacement of the talus with an alumina ceramic total talar prosthesis in anteroposterior (a) and lateral (b) views at 1 year and 6 months after surgery. There is good congruence between the talar prosthesis and the tibia, fibula, calcaneus, and navicular bone.

**Figure 5:**
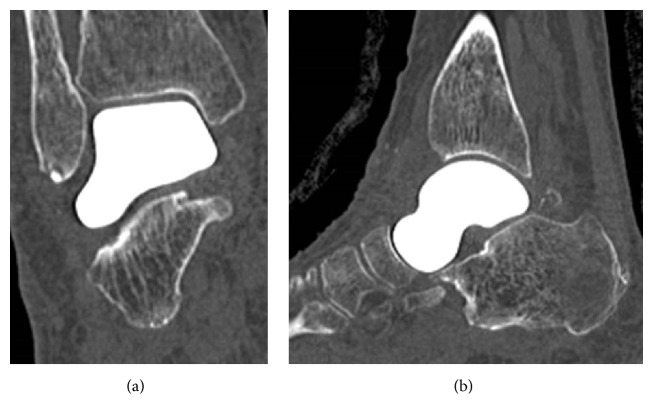
Postoperative CT scans showing good alignment of the prosthesis and surrounding bones in coronal (a) and sagittal (b) views at 1 year and 6 months after surgery. There is no osteolytic change in the surrounding bones or sinking of the prosthesis.

The ankle was immobilized in a short-leg cast for 3 weeks. The patient was allowed to resume full weight-bearing gradually and to walk as tolerated. There were no postoperative complications. Three months after surgery, she was able to mobilize while fully weight-bearing without a gait aid or pain (video 2). Examination of the right ankle showed 10° dorsiflexion and 30° plantar flexion (video 3). She was also able to stand on tiptoes (video 4)and sit square (upright), and her quality of life was substantially improved. At the most recent follow-up at 1 year and 6 months after surgery, radiographs showed that the prosthesis was appropriately positioned in the ankle with no degenerative or destructive changes in the surrounding bones (Figures 4(a) and 4(b)). There was no evidence of migration of the prosthesis on postoperative CT images (Figures 5(a) and 5(b)). JSSF ankle-hindfoot scale score had improved overall to 90 points (pain 40/40, function 40/50, and alignment 10/10). Her VAS pain score improved to 0/10.

### 2.2. Case 2

**Figure 6:**
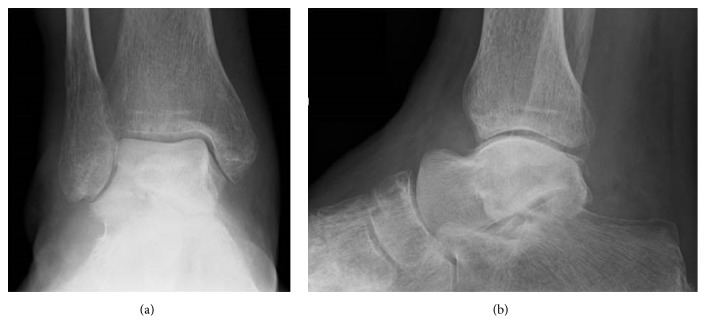
Preoperative radiographs showing sclerotic change and collapse of the talar body in anteroposterior (a) and lateral (b) views.

**Figure 7:**
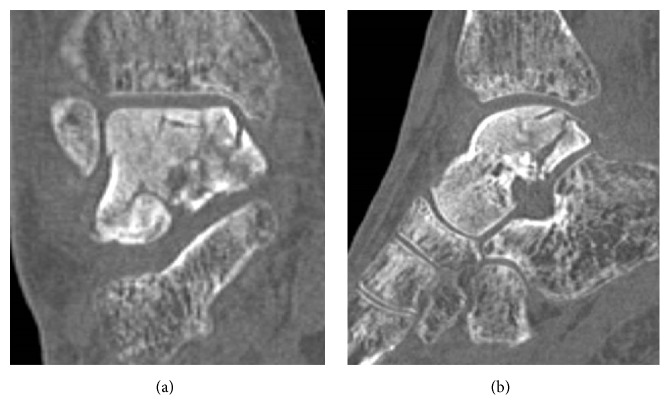
Preoperative CT showing sclerotic change and collapse of the talar body in coronal (a) and sagittal (b) views.

**Figure 8:**
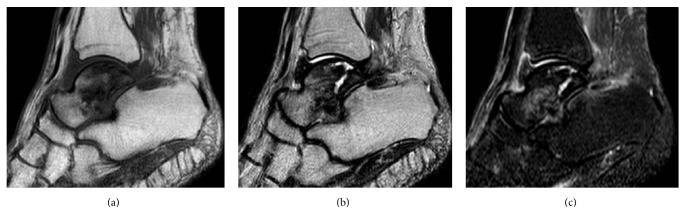
Preoperative MRI showing heterogeneous low signal intensity in the talar body on a T1-weighted image (a), a mixture of low and high signal intensity in the talar body on a T2-weighted image (b), and partial high signal intensity in the talar body on a STIR image (c).

A 78-year-old woman with hypertension and a history of cerebral infarction was referred to our department with a 3-year history of pain in the right ankle. She had no history of corticosteroid use, trauma, or alcohol abuse. She was unable to bear weight on her right lower limb. Her inability to bear weight had been progressively worsening over a period of months and she used a T cane when leaving the house. The pain in her right ankle had been worsening despite physiotherapy sessions and use of over-the-counter and prescription anti-inflammatory drugs. Physical examination revealed a limited range of motion at the right ankle (5° dorsiflexion, 30° plantar flexion), along with swelling and tenderness over the anterior aspect of the ankle. There was no distal neurovascular deficit. Her preoperative JSSF ankle/hindfoot scale score was 29/100 (pain 0/40, function 19/50, and alignment 10/10) and her VAS score for pain was 8/10. Radiography (Figures 6(a) and 6(b)) as well as CT (Figures 7(a) and 7(b)) of the right ankle showed collapse and sclerosis of the talus. MRI scans revealed an expansive talar lesion. T1-weighted images showed heterogeneous low signal intensity in the talar body (Figure 8(a)) and T2-weighted images showed a mixture of low and high signal intensity in the talar body (Figure 8(b)). STIR images showed partially high signal intensity in the talar body (Figure 8(c)). The preoperative clinical and imaging examinations suggested a diagnosis of idiopathic aseptic necrosis of the talus, and the decision was made to treat the patient using a custom-made alumina ceramic total talar prosthesis.

**Figure 9:**
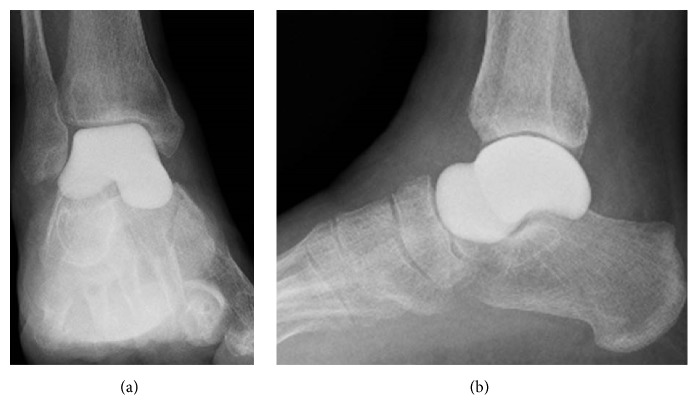
Postoperative radiographs showing replacement of the talus with an alumina ceramic total talar prosthesis in anteroposterior (a) and lateral (b) views at 1 year and 6 months after surgery. There is excellent congruence between the talar prosthesis and the tibia, fibula, calcaneus, and navicular bone.

**Figure 10:**
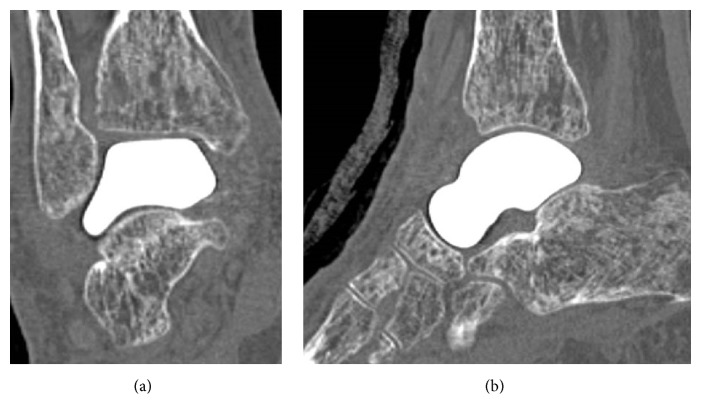
Postoperative CT scans showing that the prosthesis and surrounding bones are well aligned in coronal (a) and sagittal (b) views at 1 year and 6 months after surgery. There is no osteolytic change in the surrounding bone or sinking of the prosthesis.

Operative procedure and postoperative rehabilitation were undertaken in the same way as for case 1. There were no intraoperative or postoperative complications. At the most recent follow-up examination at 1 year and 6 months after surgery, radiographs showed that the prosthesis was in an appropriate position in the ankle with no degenerative or destructive changes in the surrounding bone tissue (Figures 9(a) and 9(b)). There was no sign of migration of the prosthesis on postoperative CT (Figures 10(a) and 10(b)). The patient was able to mobilize while fully weight-bearing without a gait aid or pain. Examination of the right ankle showed 10° dorsiflexion and 40° plantar flexion. Her JSSF ankle-hindfoot scale score improved overall to 95 points (pain 40/40, function 45/50, alignment 10/10). Her VAS pain score improved to 0/10.

## 3. Discussion

The talus is the third most common site of idiopathic osteonecrosis after the femoral head and condyle [8]. This condition is reportedly caused by heavy alcohol consumption [9], systemic lupus erythematosus [9], frequent use of corticosteroids [10, 11], or hemophilia [12, 13]. However, there was no such history in either of our cases of idiopathic necrosis of the talus.

Idiopathic talar necrosis often affects the ability to walk [14]. Tibiocalcaneal arthrodesis was introduced by Blair as a treatment for this condition [15] but has the disadvantages of loss of function and shortening of the limb [16, 17]. The procedure also requires a long recovery period and carries a considerable risk of pseudarthrosis [2, 3, 18], and a large amount of bone needs to be harvested when an iliac bone graft is used.

A talar dome prosthesis made from stainless steel was introduced by Harnroongroj and Vanadurongwan as a treatment for patients with necrosis of the talus [19]. In a subsequent report on the outcomes of talar body replacement with a metallic prosthesis by Harnroongroj and Harnroongroj [20], satisfactory function of the foot and ankle was observed during 10–36 years of follow-up. Angthong similarly reported that an anatomic metallic prosthesis provided satisfactory short-term follow-up outcomes for traumatic loss of the talus [21]. However, we are concerned about metallosis leading to failure of the talar prosthesis. Yoshinaga reported that alumina ceramic wears less than 316 L stainless steel and considered it to be an ideal material for an artificial talus [22]. We agree that alumina ceramic is a suitable material for use as the talus, where it is surrounded by articular cartilage, and opted for its use in our two cases.

The alumina ceramic talar body prosthesis was designed in Japan to replace defects in the talar body and preserve movement of the ankle [5, 6]. The first-generation implant, which had a peg on the anterior surface of the implant, was inserted into the head of the talus and fixed with bone cement. The second-generation implant was designed without a peg and was not fixed to the talar head. Both were found to have an acceptable clinical outcome but also caused a few problems. Taniguchi et al. voiced concern about loosening and sinking between the talar body prosthesis and the talar neck [6] and recommended using a third-generation prosthesis that completely replaces the talus [7]. As in our two cases, Ando et al. found that total replacement with a third-generation talar prosthesis for necrosis of the talus achieved a much better outcome in a 72-year-old woman at 2 years after surgery [23].

The advantages of total talar replacement are preservation of joint movement, a short period of restricted weight-bearing, rapid relief of pain, and preservation of limb length. Taniguchi et al. [7] and Ando et al. [23] have reported that total talar replacement with an alumina ceramic prosthesis is the most suitable treatment for necrosis of the talar body. In their cases, patients proceeded to early rehabilitation after surgery with improved range of motion at the ankle and pain-free walking. Moreover, one of our patients (case 1) was able to sit square (upright).

The potential disadvantages of total replacement of the talus are dislocation or breakage of the implant, peritalar instability, migration of the implant into the surrounding bone tissues, and osteoarthritic changes in the surrounding bone surface [23]. There were no such complications in either of our patients during 1 year and 6 months of follow-up. The long-term outcome and durability of the implant are still unknown. It should be noted that it takes 3-4 weeks to create the prosthesis because each alumina ceramic implant is individually designed using CT data from the contralateral normal side. There have been no issues of size mismatch to date. Moreover, this procedure might have a risk of the anterior instability of the talar prosthesis because the deep deltoid and anterior talofibular ligaments were divided. However, we strongly believe the residual ligaments, surrounding bones, and capsule stabilize the talar prosthesis and allowed the stability of the prosthesis, as Ando et al. reported [23].

The main limitations of this report are the small number of patients involved and lack of using a quality of life questionnaire for pathologic conditions related to the foot and ankle, such as the Self-Administered Foot Evaluation Questionnaire [24]. The other limitation of this study is short follow-up period. Therefore, additional follow-up is necessary to identify the clinical outcome of a third-generation custom-made alumina ceramic prosthesis.

In conclusion, we have successfully treated two older women who presented with aseptic necrosis of the talus using a third-generation custom-made alumina ceramic prosthesis followed by early postoperative rehabilitation. A functioning ankle joint and relief of pain were achieved in both patients. An alumina ceramic prosthesis should be considered as an alternative to an arthrodesis procedure for necrosis of the talus.

## Supplementary Material

Video 1. The patient had difficulty walking before surgery.
Video 2. The patient could walk smoothly without the aid of a crutch 3 months after surgery.
Video 3. Dorsiflexion of the affected right ankle improved from 5° to 10° and plantar flexion improved from 20° to 30° 3 months after surgery.
Video 4. The patient could stand on tiptoes of the right foot 3 months after surgery.
